# Validity between self-report and biochemical testing of cannabis and drugs among patients with traumatic injury: brief report

**DOI:** 10.1186/s42238-022-00139-8

**Published:** 2022-06-08

**Authors:** Kristin Salottolo, Emmett McGuire, Robert Madayag, Allen H. Tanner, Matthew M. Carrick, David Bar-Or

**Affiliations:** 1 Trauma Research Department, Swedish Medical Center, Englewood, 501 E Hampden Avenue, Room 4-454, Englewood, CO 80113 USA; 2 Trauma Research Department, Saint Anthony Hospital, Lakewood, CO USA; 3 Trauma Research Department, Penrose-St Francis Health Services, Colorado Springs, CO USA; 4 Trauma Research Department, Medical Center of Plano, Plano, TX USA

**Keywords:** Biochemical testing, Toxicology screen, Traumatic injury, Cannabis, Self-report validity

## Abstract

**Background:**

The relationship between drug use and traumatic injury is well documented, yet only a small proportion of patients are biochemically tested for cannabis and other substances. The study objective was to determine whether patient self-report can be used as a proxy for biochemical drug testing following traumatic injury.

**Methods:**

This study was a secondary analysis that included 320 patients admitted to four level I trauma centers in Colorado and Texas, primarily involved in motor vehicle crash (89%). If performed, biochemical testing was collected via urine toxicology screen (“tox screen”) for cannabis, amphetamines, barbiturates, cocaine, opiates, PCP, and benzodiazepines. All patients were screened for self-reported current drug use, which was evaluated for any drug and specifically for cannabis use. Analyses used to compare results of self-reported drug use and tox screen included sensitivity, specificity, positive, and negative predictive values, and percent agreement.

**Results:**

Among 320 patients, 23% (*n* = 75) self-reported drug use; cannabis was the most frequently reported drug (*n* = 63). A tox screen was performed in 59% of patients (*n* = 190); the proportion of patients who had a tox screen was similar for those self-reporting drug use (60.0%) to those who denied using drugs (59.2%), *p* = 0.90. Among patients who had a tox screen performed, 18% (*n* = 35) tested positive for any drug, 12% (*n* = 22) tested positive for THC, and 7% (*n* = 13) tested positive for opiates. The percent agreement was 80% for any drug and 81% for cannabis. The specificity was 84–85%, indicating a high likelihood that a patient will not have a positive tox screen if they do not report using drugs. Negative predictive values were 90–95%, indicating a negative self-report correctly identified nearly all patients testing negative on tox screen. Sensitivity was only 60% and positive predictive values were 30–47% for cannabis and drugs, respectively.

**Conclusion:**

These findings may negate the need for biochemical drug testing in this population, particularly as a “rule out” based on self-reporting. Future studies are needed to confirm these findings and should address risk of selection bias.

## Background

Approximately 13% of the general population aged 12 or older report current use of an illicit drug in the last month (Substance Abuse and Mental Health Services Administration 2020). In patients hospitalized with traumatic injury, approximately 30% use cannabis and other substances (Taghavi et al. 2021; Proctor et al. 2020). Moreover, the incidence of cannabis and drug use among trauma patients is increasing (Chung et al. 2019; Levine et al. 2021), potentially due to legislative efforts that have increased the availability of commercial and medical cannabis.

The American College of Surgeons mandates alcohol screening for patients admitted to the highest and most comprehensive (level I and II) trauma centers, but there are no guidelines on drug use testing in this population. A small proportion of hospitalized trauma patients are biochemically tested for drugs despite the well-established association with injury. Biochemical drug testing was performed in only 36% of patients from the National Trauma Data Bank, a registry containing nearly one million annual trauma admissions across all level I–V trauma centers (London and Battistella 2007). Elderly trauma patients are even less likely to have biochemical drug testing, reported in only 12% despite a high prevalence (48%) of a positive finding (Ekeh et al. 2014). In general, clinicians screen all trauma admissions for drug and alcohol use, but biochemical drug testing is typically reserved for patients who have high injury severity based on mechanism or vital signs or where there is a high degree of suspicion.

For alcohol, self-report is an accurate proxy for biochemical testing in a general emergency department (ED) setting (Cherpitel 2002; Vitale et al. 2006), and in patients presenting to the ED with traumatic injuries (Cherpitel et al. 2007; Sommers et al. 2000). A recent study of more than 2500 trauma patients identified inaccurate negative self-report of alcohol in just 5% of trauma patients (Hoonpongsimanont et al. 2021). Generally, there is little debate on relying on alcohol self-reporting; rather, research has shifted to what approaches further improve validity of self-report (Del Boca and Darkes 2003).

There is less consistency among studies examining the validity of self-reported drug use for biochemical testing. In ED patients seeking treatment for pain, 32% tested positive for unclaimed drugs (Schuckman et al. 2008). In Iran, nearly one in ten hospitalized patients who denied recent opioid use tested positive for opioids (Rashidian et al. 2017). On the other hand, there was little undisclosed use of drugs in outpatients diagnosed with substance use disorder (Weiss et al. 1998), and in hospitalized elderly medical patients (Glintborg et al. 2008).

It is not clear whether self-report can be used as a proxy for biochemical testing in patients presenting to the ED with traumatic injuries. The study objective was to determine the validity and agreement between patient self-report and biochemical testing following traumatic injury.

## Methods

### Study population and setting

This study was a secondary analysis of two previous retrospective studies that each collected detailed chart abstraction on self-reported drug use and biochemical drug testing via urine toxicology screen (“tox screen”). Population 1 included 254 trauma patients admitted to four level I and II trauma centers in Colorado and Texas over a period of four months (1 January 2016–30 April 2016) with motor vehicle crash (MVC) injuries. Additional information on study selection criteria can be found in previous publications (Salottolo et al. 2019; Salottolo et al. 2018). Population 2 included 66 trauma patients who were admitted to a Level I trauma center in Colorado over a period of eight months (1 March 2017–30 October 2017) for a variety of injuries. Study selection criteria for this population was previously reported (Schneider-Smith et al. 2020).

### Variables

The following variables were abstracted from the hospital’s trauma registry, which is a database collected per State requirements for reporting, quality improvement, and quality assurance: admission date and time; tox screen results (positive, negative, not tested); age in years; sex; cause of injury (MVC, fall, other injury); injury severity score (ISS; range 0–75, values ≥ 16 denote severe traumatic injury); ED Glasgow coma score (GCS; range 3–15, values 3–8 denote severe head injury and values of 15 denote normal neurologic exam); hospital LOS in days. Dedicated trauma research coordinators abstracted information from the patient’s electronic medical record (EMR) on self-reported drug use and, in some cases, specific findings from the tox screen if they were not recorded in the registry.

If performed, the tox screen was ordered by the clinician (typically the admitting trauma service) and collected on admission, after the history and physical (H&P) examination. Tox screens were performed using the Siemens Vista® 1500 instrument. The urine threshold levels for each drug are as follows: amphetamines (1000 ng/mL); barbiturates (200 ng/mL); Benzodiazepines (200 ng/mL); tetrahydrocannabinol (THC, 50 ng/mL); cocaine (300 ng/mL); MDMA (500 ng/mL); opiates (300 ng/mL). The drug tox screen was considered positive if any of the above drugs were detected. Cannabis use was separately examined and defined as positive when THC was detected.

All patients are screened for self-reported drug use, which was documented within the EMR by reviewing the Emergency Medical Service report, Screening, Brief Intervention, and Referral to Treatment (SBIRT) documentation, H&P, and progress notes. Self-reported drug use was examined as the use of any illicit/illegal drug; we separately examined cannabis.

Statistical analysis was performed using SAS version 9.4 (SAS®, Cary, NC). Analyses used to compare self-reported drug use and tox screen findings included measures of validity (sensitivity, specificity, positive and negative predictive values) and percent agreement. There was no threshold for statistical significance and no formal power calculations were performed.

## Results

The analysis included 320 trauma patients. The median (interquartile) age of the population was 35 (24–55) years, 63% were male, with mild to moderate trauma (median ISS of 10), 82% presented with no neurologic deficits (GCS 15), and the median LOS was 4 (2–8) days. The majority were injured in an MVC (89%): all patients in population 1 were injured in an MVC, compared to population 2, where 47% were injured in an MVC, 32% were injured from a fall, and 21% had another cause of injury.

Self-reported drug use was identified in 23% of patients (*n* = 75). Of these, 63 patients self-identified as cannabis users.

Tox screens were performed in 59% of patients; the proportion of patients who had a tox screen was similar for those who self-reported drug use (60.0%) compared to patients who did not self-report using drugs (59.2%), *p* = 0.90. Likewise, the proportion of patients with a tox screen was similar by patients self-reporting cannabis use or not (60.3% vs. 59.1%, *p* = 0.87).

Among the 59% (*n* = 190) patients who had a tox screen performed, 18% (*n* = 35) tested positive for any drug and 12% (*n* = 22) tested positive for THC. Besides cannabis, the most common drug identified was opiates (7%, *n* = 13). Compared to patients without a tox screen, those tox screened were similar demographically (median age, *p* = 0.98; % male, *p* = 0.27), and by injury severity (% GCS 15, *p* = 0.27; median ISS, *p* = 0.06), but were more likely to be injured by MVC (98% vs. 76%) and less likely to be injured due to a fall (2% vs. 14%), *p* < 0.001.

The validity of self-reported drug use for tox screen results is shown in Table 1. The percent agreement was 80% for any drug and 81% for cannabis. A negative self-report of drug use correctly identified 90% of patients who tested negative for drugs. A negative self-report of cannabis correctly identified 95% of patients who tested negative for THC. Specificity of 84–85% indicated a high chance that a patient did not test positive for THC/drugs if the patient did not self-report use.

**Table 1:** Agreement between self-reported drug use and biochemical testing via urine toxicology screen for 190 patients admitted with traumatic injury

	Any drug		Cannabis	
Response	Tox +	Tox –	Tox +	Tox –
Self-report—yes	21	24	12	28
Self-report—no	14	131	8	142
Sensitivity (95% CI)	0.60 (0.42, 0.76)		0.60 (0.36, 0.81)	
Specificity (95% CI)	0.85 (0.78, 0.90)		0.84 (0.77, 0.89)	
PPV (95% CI)	0.47 (0.32, 0.62)		0.30 (0.17, 0.47)	
NPV (95% CI)	0.90 (0.84, 0.95)		0.95 (0.90, 0.98)	

*Tox* urine toxicology screen, *PPV* positive predictive value, *NPV* negative predictive value

Results are tabulated for the subset of patients with toxicology screening. Patients were defined as being tox screen positive (Tox +) or negative (Tox−) based on detection on the multi-drug panel (amphetamines, barbiturates, benzodiazepines cocaine, opiates, MDMA, and tetrahydrocannabinol (THC; cannabis)

Sensitivity was 60% for any drug as well as 60% for cannabis. Positive predictive values were 47% for any drug but only 30% of patients who self-reported cannabis use tested positive for THC.

## Discussion

This secondary analysis sought to determine the agreement between a trauma patient’s self-reported drug use and biochemical drug testing via urine tox screen. These findings suggest that in patients with traumatic injury, self-report is a valid proxy for ruling out drug use, and there were no apparent differences in the validity of using self-report for cannabis compared to other drugs.

There are several advantages to being able to rely on self-reported drug use, such as immediate results, no cost, and noninvasiveness. Self-report is also incentivized by hospitals because insurance companies in some states are allowed to deny reimbursement if an injury resulted from alcohol and drug use (Elkbuli et al. 2019).

However, there are clinical concerns with undisclosed drug use for patients presenting acutely to the ED. Drug use can confound vital sign assessments, diagnosis, and treatment (DiGiorgio et al. 2020), there are implications for drug-drug interactions (Dydyk et al. 2020), and risk for withdrawal (Arroyo-Novoa et al. 2020; Jawa et al. 2014; Salottolo et al. 2017), and there are negative effects on acute pain management (Salottolo et al. 2019; Salottolo et al. 2018) and clinical outcomes (Taghavi et al. 2021). Because of these concerns with undisclosed drug use, there is frequent debate about biochemically testing trauma patients for drugs. Our experience suggests clinicians are hesitant to rely on self-report, instead requesting a tox screen to determine if drugs are present. Tox screens were performed in 59% of trauma patients in this study, in the range reported in the literature of 36% (London and Battistella 2007) to 85% (Grigorian et al. 2019).

While these findings suggest there is utility in using self-reported drug use in trauma patients, as undisclosed use was rare for cannabis and other drugs, the implications of our findings should be considered with the study size and setting. Only 190 of 320 trauma patients had biochemical drug testing, and most of our population was admitted to hospitals in Colorado, the first state to legalize and commercialize cannabis. Patients in Colorado may be more willing to divulge drug use, especially cannabis. Currently, 37 states plus the District of Columbia, Guam, and the US Virgin Islands have permissive cannabis laws legalizing medical cannabis (National Conference of State Legislatures 2021), but our findings may not be generalizable to states with strict marijuana laws where medical and recreational cannabis are illegal. Published studies demonstrate an increase in self-reported cannabis use after medical legalization (Levine et al. 2021) and recreational legalization (Grigorian et al. 2019; Jennings et al. 2019), although one study reported a decrease in the percent of patients self-reporting use after medical marijuana legalization (Claudius et al. 2020).

Vitale et al. recommend using drug self-report in patients presenting to the ED for injuries or illness because it provides more accurate information than the ‘gold standard’ of biochemical testing for both alcohol and drugs (Vitale et al. 2006). In our study, sensitivity and positive predictive values were poor, suggesting that a negative tox screen may not accurately recognize a patient who is a current drug user. The sensitivity of cannabis was 60% in our study. Similarly, Claudius et al. reported 53% of trauma patients testing positive for cannabis also self-reported use (Claudius et al. 2020). In a roadside survey in Belgium, self-reported cannabis use was compared with biochemical testing in nearly 3000 drivers; there was high specificity (94–99%) but low sensitivity (22–58%) between self-report and biochemical testing (Van der Linden et al. 2014).

This study has limitations. Primarily, tox screens are not routinely ordered on all trauma patients or on a random set of patients. Results would be more interpretable in a setting with universal biochemical testing. Tox screens are typically ordered in the trauma setting based on clinical relevance (e.g., differential diagnosis or in treatment) and injury characteristics (e.g., severity, cause, or mechanism). In some settings, tox screens are ordered on clinical suspicion or to confirm drug usage. In our study, the proportion of patients who had a tox screen were statistically identical by self-reported drug use vs. non-use (60% vs. 59%). Demographic characteristics were also similar based on whether a tox screen was done. There were differences based on cause of injury: tox screens were more frequently ordered for patients with a MVC injury. Had there been differences in tox screen testing based on self-reported drug use or demographics, this would have been a major source of selection bias. Fortunately, this was not observed. Still, future studies should test all patients or a random sample of patients in order to confirm our findings. A related limitation is that the statistical analyses were limited to the 59% of patients who had a tox screen. Based on prior studies at U.S. trauma centers, biochemical testing is ordered for 36 to 85% of trauma admissions, similar to what we are reporting.

Additional limitations are as follows. Third, this was a post hoc analysis of two convenience samples, and the populations differed in age, ISS, and cause of injury. Fourth, patients who were aware their urine was being collected for biochemical testing may have also been more willing to divulge illicit drug use, despite the risk of retribution or penalty. However, our data do not support this possibility since self-reported drug use was reported in 23.1% without a tox screen and 23.7% who had a tox screen (*p* = 0.90). Fifth, patients who were immediately treated with opiates or other benzodiazepines may have been misclassified as a positive tox screen, because urine sample collection is generally performed on admission and not in the ED. Finally, it is possible that the tox screen was negative because the drug was not present in detectable concentrations at the time of hospital admission. Approximate drug detection times in urine vary based on drug (Moeller et al. 2017). Alternatively, synthetic “designer” drugs, which are increasingly used, although still rare at only 1.2% of teens and young adults (Palamar et al. 2015), may be undetected in routine urine tox screens (Luethi and Liechti 2020).

## Conclusion

This exploratory analysis of 320 trauma patients suggests excellent validity in using self-report for ruling out drug use including cannabis use, affirming that a patient is not a user if they deny use. Undeclared use of cannabis was especially rare. These findings also underscore the issues in relying on biochemical testing as the gold standard for drug use.

## Data Availability

The datasets used and/or analyzed during the current study are available from the corresponding author on reasonable request.

## Abbreviations

ED: Emergency department
EMR: Electronic medical record
GCS: Glasgow coma score
ISS: Injury Severity Score
LOS: Length of stay
MVC: Motor vehicle crash
PCP: Phencyclidine
SBIRT: Screening, Brief Intervention, and Referral to Treatment
THC: Tetrahydrocannabinol.
